# Bisphenol AF-Induced Endogenous Transcription Is Mediated by ERα and ERK1/2 Activation in Human Breast Cancer Cells

**DOI:** 10.1371/journal.pone.0094725

**Published:** 2014-04-11

**Authors:** Ming Li, Jing Guo, Wenhui Gao, Jianlong Yu, Xiaoyu Han, Jing Zhang, Bing Shao

**Affiliations:** 1 Beijing Key Laboratory of Diagnostic and Traceability Technologies for Food Poisoning, Beijing Center for Disease Control and Prevention, Beijing, China; 2 Department of Emergency, Beijing Mentougou District Hospital, Beijing, China; 3 School of Public Health and Family Medicine, Capital Medical University, Beijing, China; II Università di Napoli, Italy

## Abstract

Bisphenol AF (BPAF)-induced transcriptional activity has been evaluated by luciferase reporter assay. However, the molecular mechanism of BPAF-induced endogenous transcription in human breast cancer cells has not been fully elucidated. In the present study, we investigated the effect and mechanism of BPAF-induced endogenous transcription detected by real-time PCR in human breast cancer cells. We found that BPAF stimulated transcription of estrogen responsive genes, such as trefoil factor 1 (*TFF1*), growth regulation by estrogen in breast cancer 1 (*GREB1*) and cathepsin D (*CTSD*), through dose-dependent and time-dependent manners in T47D and MCF7 cells. Gene-silencing of ERα, ERβ and G protein-coupled estrogen receptor 1 (GPER) by small interfering RNA revealed that BPAF-induced endogenous transcription was dependent on ERα and GPER, implying both genomic and nongenomic pathways might be involved in the endogenous transcription induced by BPAF. ERα-mediated gene transcription was further confirmed by inhibition of ER activity using ICI 182780 in ERα-positive T47D and MCF7 cells as well as overexpression of ERα in ERα-negative MDA-MB-231 breast cancer cells. Moreover, we utilized Src tyrosine kinase inhibitor PP2 and two MEK inhibitors PD98059 and U0126 to elucidate the rapid nongenomic activation of Src/MEK/ERK1/2 cascade on endogenous transcription. Our data showed that BPAF-induced transcription could be significantly blocked by PP2, PD98059 and U0126, suggesting activation of ERK1/2 was also required to regulate endogenous transcription. Taken together, these results indicate that BPAF-induced endogenous transcription of estrogen responsive genes is mediated through both genomic and nongenomic pathways involving the ERα and ERK1/2 activation in human breast cancer cells.

## Introduction

During the past two decades, bisphenol A (BPA) and its analogues have received growing concerns for their endocrine disrupting properties and ubiquitous occurrences [1], [2]. Bisphenol AF (BPAF) is a bisphenol analogue of BPA and primarily used as a monomer for polyimides, polyamides, polyesters and other specialty polymers and as a cross linker for certain fluoroelastomers [3]. BPAF as an EDC has been nominated for its toxicological characterization by National Toxicology Program, National Institute of Environmental Health Sciences considering the potential exposure of the general population and the similar structure to BPA [4]. It has been reported that BPAF is extensively occurred in the river, sediment and soil after released into the ambient environment around a manufacturing plant in China [5].

BPAF has been shown to induce estrogenic actions *via* binding to estrogen receptor (ER) [6], [7]. BPAF strongly binds to both ERα and ERβ as detected by radioligand binding assay. The receptor-binding activity of BPAF is about three times more potent for ERβ than for ERα [8]. Luciferase reporter assay indicates that BPAF is a full agonist for ERα, but displays little potency to activating ERβ. However, BPAF shows antagonist activity on ERβ as against 17β-estradiol (E2) in HeLa cells [8]. Moreover, BPAF was also reported as an agonist of human pregnane X receptor, which may contribute to BPAF-induced adverse effects in human [9].

Regulation of estrogenic effects is a multifactorial and complex process, involving both genomic and nongenomic actions. In the genomic action, ERα regulates gene expression *via* directly binding to estrogen responsive element (ERE) or by interacting with other transcription factors, such as AP1 and Sp1 [10]–[12]. In the nongenomic action, G protein-coupled estrogen receptor 1 (GPER, also known as GPR30) mediates the activation of signaling cascades involving extracellular signal regulated kinase 1 and 2 (ERK1/2), Src tyrosine kinase and Akt [13]. However, ERα has also been implicated in E2-stimulated rapid activation of ERK1/2 in ERα transfected COS7 and HEK293 cells [14].

Trefoil factor 1 (*TFF1*), also known as *pS2*, is a well-studied estrogen responsive gene. There are three ERα binding sites located at approximately −0.3 kb, −9.1 kb and −9.9 kb in 5′-flanking region of the *TFF1* gene. Growth regulation by estrogen in breast cancer 1 (*GREB1*) plays an important role in the regulation of proliferation of breast cancers. Three EREs are spread over approximately 20 kb of promoter region of *GREB1*. Expression of cathepsin D (*CTSD*), which is a lysosomal protease, could be mediated by ERα binding to ERE sites located at 9 and 33 kb upstream of the transcription start site. BPAF-induced estrogenic activity, which was commonly detected by ERE-luciferase reporter, has been widely reported [7], [9], [15]. However, little is known about the mechanism of BPAF-induced endogenous transcription which could influence many physiological processes both *in vitro* and *in vivo*. In the present study, we evaluated the effect of BPAF on endogenous transcription of *TFF1*, *GREB1* and *CTSD* in human breast cancer cells. To better understand the potential mechanism, we investigated the role of both genomic and nongenomic actions on BPAF-stimulated endogenous transcription.

## Material and Methods

### Materials

BPAF was purchased from Tokyo Chemical Industry Co., Ltd. (Tokyo, Japan), E2 was purchased from Dr. Ehrenstorfer GmbH (Germany), and ICI 182780 was obtained from Ascent Scientific. Negative control small interfering RNAs (siRNA) (Ambion #4390846), ERα siRNA (Ambion #4823), ERβ siRNA (Ambion #4826) and GPER siRNA (Ambion #6053) were purchased from Life Technologies (Beijing, China). PP2, PD98095 and U0126 were purchased from Sigma (Shanghai, China).

### Cell culture

T47D, MCF7 and MDA-MB-231 human breast cancer cell lines were purchased from Cell Bank (Shanghai Institute of Biochemistry and Cell Biology, Chinese Academy of Sciences) and cultured in RPMI-1640 medium supplemented with 10% fetal bovine serum (FBS) (Gibco) at 37°C and 5% CO_2_/95% air. When the cells grew to 70% confluence, the culture medium was changed to RPMI-1640 supplemented with 10% charcoal-stripped FBS (SERANA, Australia) for 3 days before treatment in order to minimize the estrogen activity from serum. 293AD cells for generating adenovirus were kindly provided by Dr. Yulia Nefedova at Wistar Institute, and cultured in DMEM medium supplemented with 10% FBS.

### RNA extraction and real time-PCR

Total RNA was extracted using the RNeasy Mini Kit (QIAGEN) according to the manufacture protocol. 1 µg total RNA was reverse-transcribed using Superscript reverse transcriptase (Promega). The mRNA levels of estrogen responsive genes and control gene GAPDH were measured by quantitative real-time PCR using Power SYBR Green PCR Master Mix (Applied Biosystems). Primers for *TFF1* were forward 5′-GTGCAAATAAGGGCTGCTGTT-3′ and reverse 5′-CACACTCCTCTTCTGGAGGGA-3′, primers for *GREB1* were forward 5′-ATTGGTGGACCGATTGCTCA-3′ and reverse 5′-GCTGATGAGGGTGTGCTGTGT-3′, primers for *CTSD* were forward 5′-CAGCCAGGCATCACCTTCAT-3′ and reverse 5′-CAGGTAGAAGGAGAAGATGT-3′ and primers for *GAPDH* were forward 5′-CACCCACTCCTCCACCTTTGA-3′ and reverse 5′-ACCACCCTGTTGCTGTAGCCA-3′. The reactions were performed in an ABI Prism 7300 Sequence Detection system for 40 cycles (95°C for 15 sec, 60°C for 60 sec) after an initial 10 min incubation at 95°C. The fold change in the expression of *TFF1*, *GREB1* and *CTSD* was calculated using 2^−ΔΔCt^ method. Each sample was assayed in triplicate.

### siRNA transfection assay

T47D and MCF7 cells were seeded in 12-well plates at the density of 2×10^5^ cells per well and transfected with negative control, ERα, ERβ or GPER siRNA at a final concentration of 50 nmol/L with lipofectamin RNAiMAX reagent (Life technologies) according to the manufacturer's instruction. Medium was changed 3 h after transfection, and BPAF treatments were initiated after another 24 h. Cells were subjected to western blotting to detect protein expression at 48 h after siRNA transfection.

### Western blotting analysis

Cells were harvested and lysed in RIPA lysis buffer supplemented with protease inhibitor cocktail (Sigma) and phosphatase inhibitor cocktail (Sigma). The cell lysates were incubated on ice for 15 min and centrifuged at 12,000 g at 4°C for 15 min. Protein concentration was determined by Bradford protein assay kit (Bio-Rad Laboratories). Equivalent amount of protein was resolved by 10% SDS-PAGE and transferred to polyvinylidene difluoride membranes. The membranes were blocked with 5% BSA in TBST (10 mM Tris-HCl, pH 8.0, 150 mM NaCl and 0.1% Tween 20) for 1 h, then incubated with primary antibodies overnight at 4°C. The primary antibodies against the following proteins were used: anti-ERα (Cell Signaling Technology.), anti-ERβ (Sant Cruz), anti-GPER (Sant Cruz) and anti-β-actin (Cell Signaling Technology). After three washes for 10 min each time in TBST, membranes were incubated with horseradish peroxidase-conjugated secondary antibodies for 1 h, and detected using Pierce ECL plus detection kit (Thermo Scientific).

### Construction of adenovirus vectors and infections

The cDNA sequence encoding full-length human ERα was PCR-amplified with the forward primer 5′-ACCGGTACCAGCCACCATGACCATGACCCT-3′ and reverse primer 5′-TTGGAATTCTCAGACCGTGGCAGGGA-3′ from the pGV227-ESR1 clone purchased from Genechem. The amplified PCR product was inserted into the pacAd5 CMV KNpA vector (Cell Biolabs) after digestion with *Kpn*I and *EcoR*I, generating the pacAd5-ERα vector. PacAd5-GFP as control was provided by Cell Biolabs. Recombinant adenoviral genome encoding human ERα or GFP was generated by homologous recombination and expanded in 293AD cells using standard protocol (Cell Biolabs). High-titer adenovirus preparations were obtained as described [16]. The final viruses were stored at −80°C in phosphate-buffered saline with 10% glycerol. The titers of the viruses were determined by infecting 293AD cells at various dilutions using Adeno-X rapid titer kit (Clontech). The functional titers of Ad-ERα and Ad-GFP stocks were 5.56×10^10^ ifu/mL and 4.3×10^10^ ifu/mL, respectively.

Before adenovirus infections, the cell culture medium was change to RPMI-1640 medium without FBS. MDA-MB-231 cells were infected with adenovirus at a multiplicity of infection (MOI) of 300 for 15 min in 12-well plates (2×10^5^ cells per well). Then, the medium containing virus was changed to the fresh complete medium.

### Statistical analysis

All experiments were repeated at least three times. Results are expressed as mean ± S.E.M. Statistical analysis was performed using Student's *t*-test. A *P* value <0.05 was considered statistically significant.

## Results

### Effect of BPAF on endogenous transcription of estrogen responsive genes

**Figure 1:**
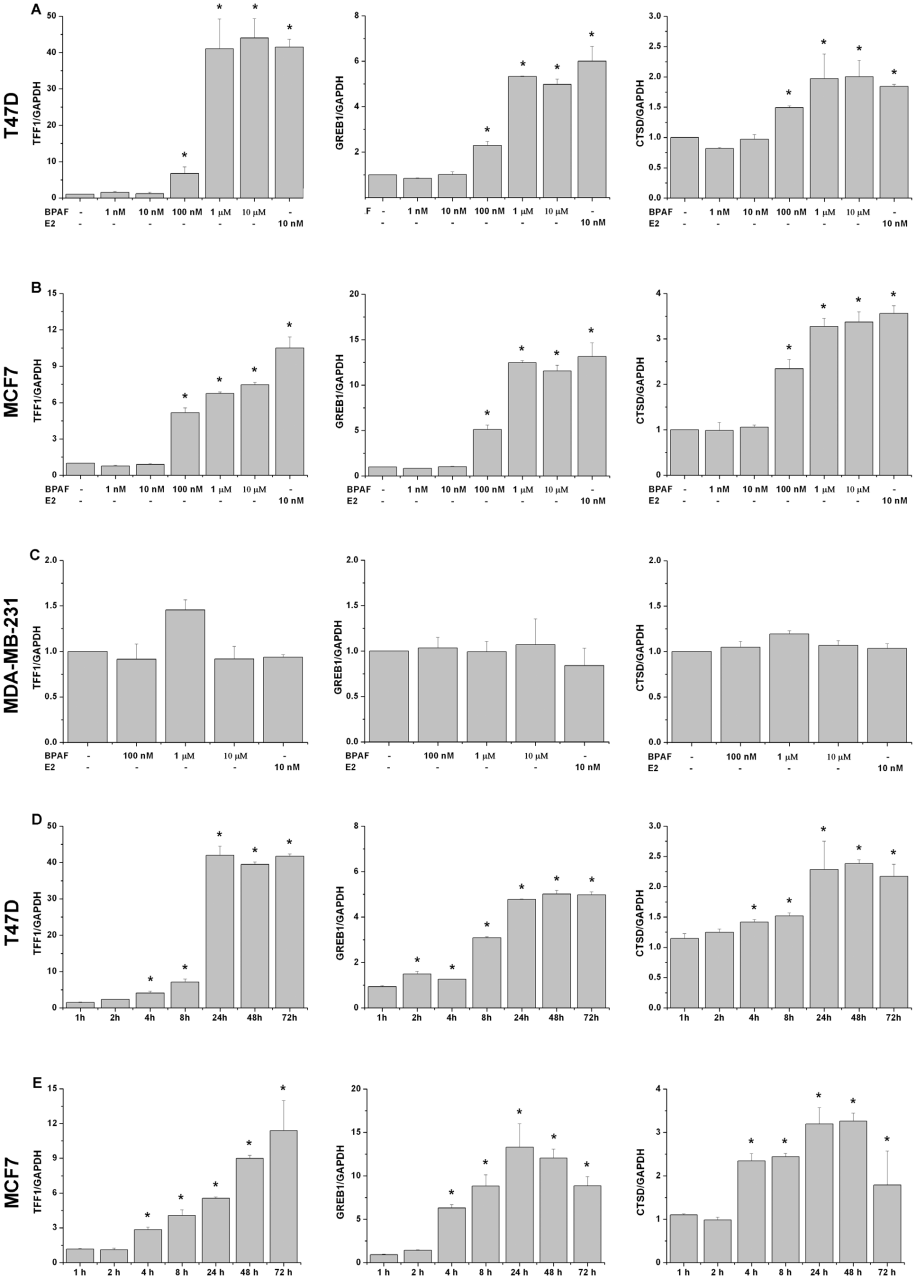
Effect of BPAF on endogenous transcription of *TFF1*, *GREB1* and *CTSD* in human breast cancer cells. Dose-dependent response to BPAF on endogenous transcription of *TFF1*, *GREB1* and *CTSD* in T47D (A), MCF7 (B) and MDA-MB-231 (C) cells. Cells were treated with BPAF at indicated concentrations for 24 h. *P<0.05, compared to the control. Time-dependent response to BPAF on endogenous transcription of *TFF1*, *GREB1* and *CTSD* in T47D (D) and MCF7 (E) cells. Cells were treated with 1 µM BPAF for various time points. *P<0.05, compared to the vehicle control at the indicated time point. Data shown represent results from three independent experiments.

To evaluate the effect of BPAF on endogenous transcription, we conducted the real-time PCR to detect mRNA levels of estrogen responsive genes, such as *TFF1*, *GREB1* and *CTSD* in ERα-positive T47D and MCF7 breast cancer cell lines and ERα-negative MDA-MB-231 breast cancer cell line. In both T47D (Figure 1A) and MCF7 (Figure 1B) cells, BPAF significantly induced *TFF1*, *GREB1* and *CTSD* mRNA expression at the concentrations of 100 nM–10 µM (*P*<0.05) in a dose-dependent manner compared with controls, but the induction was not observed even at the concentration of 100 µM due to the strong toxicity of BPAF (data not shown). The maximal transcription was achieved at 1 µM, which was comparable with 10 nM E2 as positive control. In contrast, BPAF did not induce *TFF1*, *GREB1* and *CTSD* mRNA expression in MDA-MB-231 cells (Figure 1C).

Next, we performed a time course analysis of *TFF1*, *GREB1* and *CTSD* transcription stimulated by 1 µM BPAF in T47D and MCF7 cells. Our data showed that BPAF significantly enhanced the transcription of ER-regulated genes after 4 h treatment (*P*<0.05) and in a time-dependent manner for up to 24 h. The maximum mRNA levels were maintained during the later period of treatment in T47D cells. For MCF7 cells, BPAF-induced *TFF1* expression exhibited a time-dependent manner during the whole experiment period, whereas the induction of *GREB1* and *CTSD* at 72 h was obviously lower than that at 24 h and 48 h. According to the above results, BPAF could stimulate endogenous transcription of *TFF1*, *GREB1* and *CTSD* through time-dependent manners within 24 h. Then, we investigated the mechanism of BPAF-induced endogenous transcription in human breast cancer cells treated with 1 µM BPAF for 24 h.

### BPAF-induced endogenous transcription was mediated by ERα and GPER

**Figure 2:**
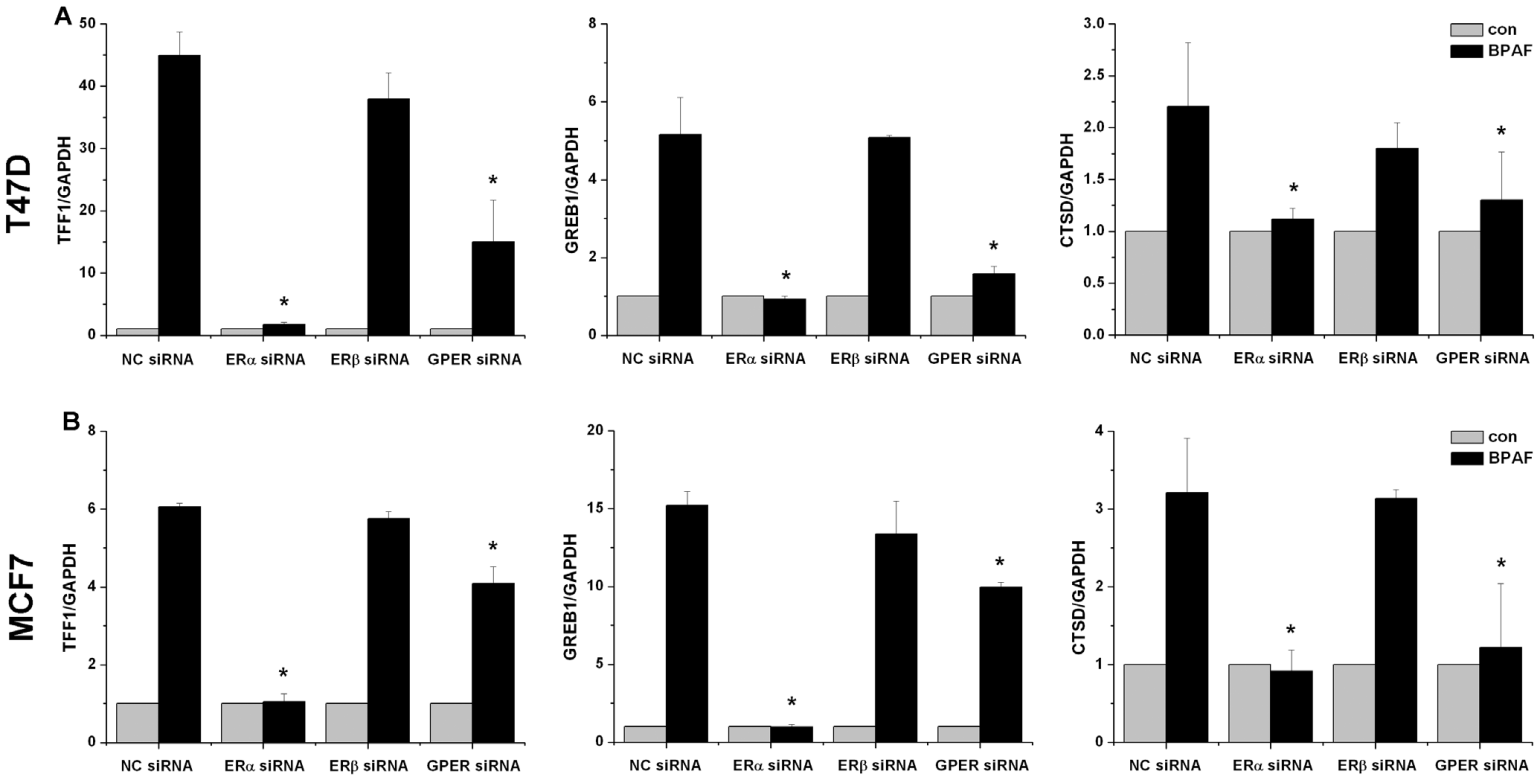
Effect of knockdown of ERα, ERβ or GPER on BPAF-induced endogenous transcription in T47D (A) and MCF7 (B) human breast cancer cells. T47D and MCF7 cells were transiently transfected with negative control (NC), ERα, ERβ or GPER siRNA for 24 h, then treated with vehicle alone or 1 µM BPAF for 24 h. Data shown are representative from at least three independent experiments. *P<0.05, compared to BPAF-stimulated cells transfected with NC siRNA.

To explain the mechanism of BPAF-induced endogenous transcription, siRNA against ERα, ERβ and GPER was used in T47D and MCF7 cells. The protein and mRNA levels of ERα, ERβ and GPER were decreased significantly after siRNA transfection (Figure S1 in File S1). Our data showed that transfection of T47D (Figure 2A) and MCF7 (Figure 2B) cells with ERα or GPER siRNA significantly inhibited the endogenous transcription in response to BPAF compared to the negative control (NC) siRNA (*P*<0.05). In contrast, silencing of ERβ did not affect BPAF-induced endogenous transcription.

**Figure 3:**
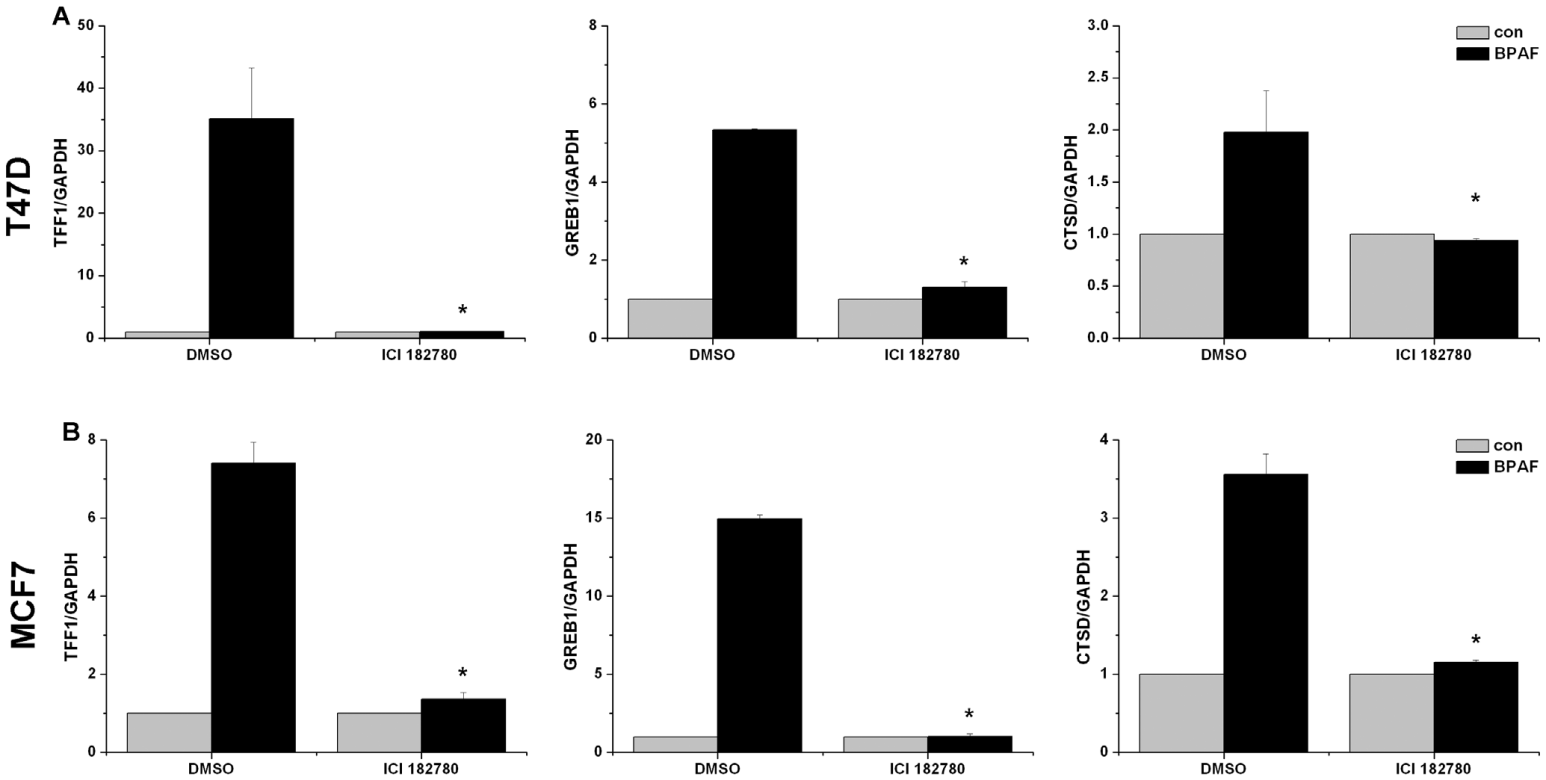
Antiestrogen ICI 182780 inhibits BPAF-induced endogenous transcription in T47D (A) and MCF7 (B) cells. T47D and MCF7 cells were co-treated with 1 µM ICI 182780 or DMSO in the presence or absence of 1 µM BPAF for 24 h. Data shown are representative from at least three independent experiments. *P<0.05, compared to BPAF-stimulated cells in the present of DMSO as vehicle control.

**Figure 4:**
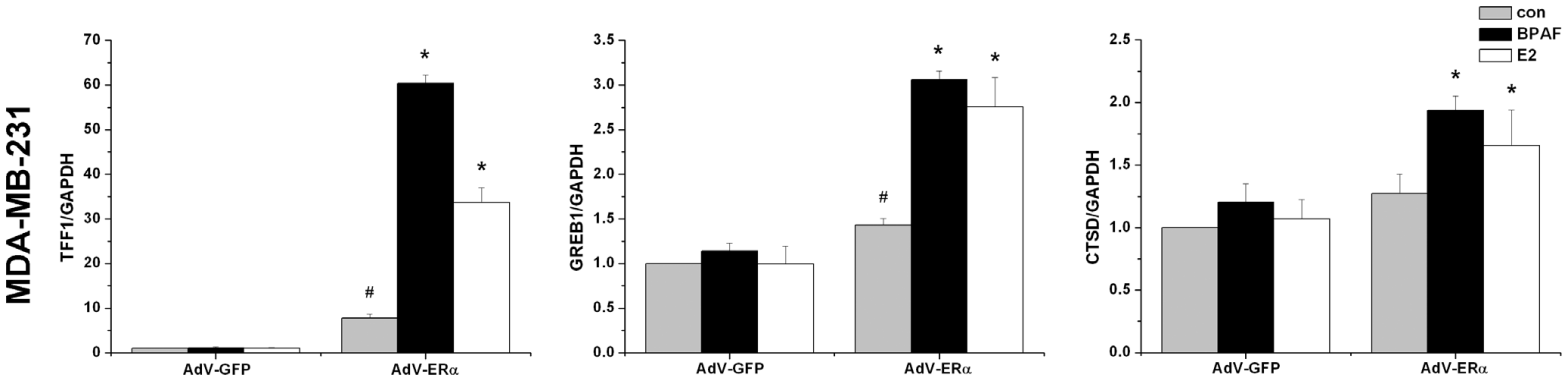
Overexpression of ERα increased BPAF-induced endogenous transcription in MDA-MB-231 human breast cancer cells. MDA-MB-231 cells were infected with adenovirus expressing the ERα (Ad-ERα) and GFP (Ad-GFP) for 15 min, respectively. After 24 h incubation, MDA-MB-231 cells were treated with 1 µM BPAF or 10 nM E2 as positive control for 24 h. Data shown are representative from three independent experiments. *P<0.05, compared to BPAF-stimulated MDA-MB-231 cells infected with AdV-GFP. #*P*<0.05, compared to MDA-MB-231 cells infected with AdV-GFP.

To verify the role of ER in endogenous transcription, antiestrogen ICI 182780 was used to inhibit both ERα and ERβ activities. BPAF-induced transcription was significantly decreased in the presence of 1 µM ICI 182780 in both T47D (Figure 3A) and MCF7 (Figure 3B) cells (*P*<0.05), indicating that BPAF-induced gene transcription was mediated by ER activity, but we could not distinguish which receptor mediates the transcription. However, the induction of *TFF1*, *GREB1* and *CTSD* did not occur in the ERα-negative MDA-MB-231 cells. In addition, ERβ knockdown did not affect BPAF-induced transcription in T47D and MCF7 cells, suggesting that the ER involved is ERα. To further confirm the function of ERα, we constructed adenoviruses for overexpression of ERα in MDA-MB-231 cells, which was detected by western blot and real-time PCR (Figure S2 in File S1). mRNA levels of *TFF1*, *GREB1* and *CTSD* were induced significantly by BPAF or E2 as positive control in MDA-MB-231 cells with overexpression of ERα (Figure 4) (*P*<0.05). Moreover, overexpression of ERα in MDA-MB-231 cells without BPAF treatment also could significantly stimulate the transcription of *TFF1* and *GREB1* but not *CTSD* compared to that of GFP (*P*<0.05). Based on the siRNA, inhibitor and adenovirus-mediated transduction to regulate the protein level and activity of ERα in breast cancer cells, our results suggested that ERα is responsive for BPAF-induced endogenous transcription.

Of note, knocking down GPER blocked BPAF-stimulated transcription, indicating GPER-mediated nongenomic pathway is also involved in regulation of endogenous transcription in breast cancer cells. Therefore, we investigated the role of nongenomic action on BPAF-induced endogenous transcription.

### BPAF-induced endogenous transcription was dependent on ERK1/2 activation

**Figure 5:**
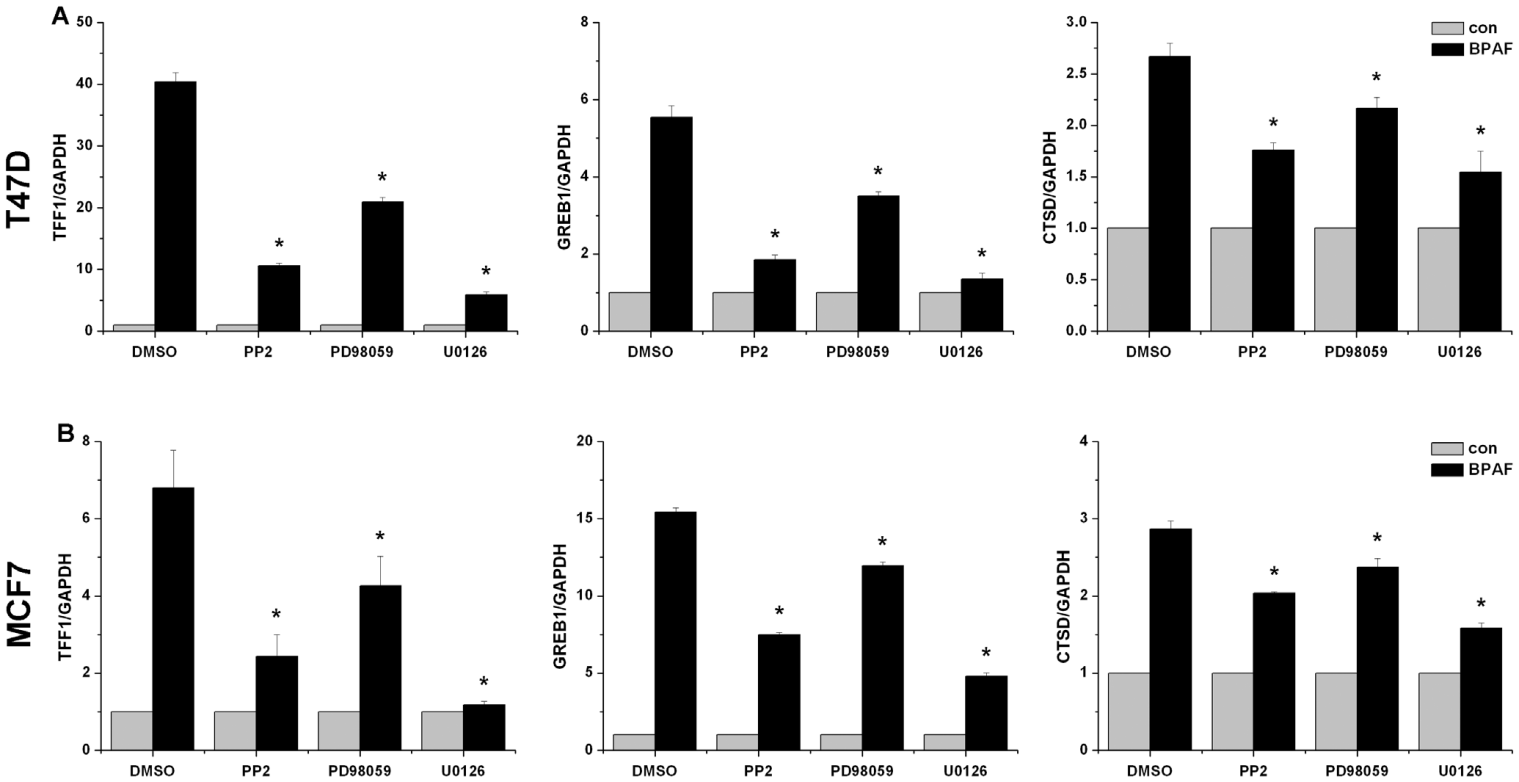
Activation of Src/MEK/ERK1/2 is required for BPAF-induced endogenous transcription. T47D (A) and MCF7 (B) cells were treated with 10 µM PP2, 2 µM PD98095 or 50 µM U0126 for 1 h, then incubated in the presence or absence of 1 µM BPAF for 24 h. Data shown represent results from three independent experiments. *P<0.05, compared to BPAF-stimulated cells in the present of DMSO as vehicle control.

In the nongenomic action, Src/MEK/ERK1/2 signaling was rapidly activated by estrogen in hormone sensitive cells [17]. We observed that ERK1/2 could be phosphorylated at 15 min following BPAF stimulation in both T47D and MCF7 cells (Figure S3 in File S1), which is consistence with Li *et al*'s report [6]. Then, we examined the effects of Src inhibitor PP2 and two MEK inhibitors PD98095 and U0126 on BPAF-induced transcription of estrogen responsive genes in T47D (Figure 5A) and MCF7 (Figure 5B) cells. We found that BPAF-mediated mRNA expression of *TFF1*, *GREB1* and *CTSD* was significantly decreased by treatment of cells with PP2, PD98095 and U0126, respectively (*P*<0.05). These findings suggested that BPAF-induced activation of ERK1/2 was also required for endogenous transcription of estrogen responsive genes.

## Discussion

Various *in vitro* assays have been established for evaluating estrogenic activity of xenoestrogen, such as reporter gene assay, yeast-based reporter assay, E-screen assay based on cell proliferation [18]–[20]. However, these assays can not reflect the effect of xenoestrogen on the endogenous transcription, which influence the endocrine functions in the cellular and animal models. In the present study, we investigated BPAF-induced endogenous transcription of estrogen responsive genes in human breast cancer cells. We found BPAF stimulated the endogenous transcription of *TFF1*, *GREB1* and *CTSD* genes through both dose and time-dependent manners in ERα-positive T47D and MCF7 human breast cancer cells, but not in ERα-negative MDA-MB-231 cells. We observed that endogenous transcription could be induced by 100 nM BPAF, and reach to maximum at 1 µM. However, it has been reported that BPAF exhibited estrogenic activity with the EC_50_ of 50 nM using the ERE reporter assay in MCF7 cells [21]. BPAF could stimulate ERE luciferase reporter even at 10 nM in ER-negative cell lines co-transfected with ERE-luciferase reporter and wild type-ERα [6]. Compared to the reporter assay, the less sensitivity of real-time PCR assay may be as a result that more transcription factors and additional elements besides the ERE are required for the endogenous transcription.

The development of estrogen responsive tumors could be modulated by ER through controlling transcription of various genes thereby influencing tumor phenotype [22], [23]. Approximately 70% of all human breast cancers are ERα-positive and dependent on hormones for proliferation. Therefore, understanding the estrogenic activity of environmental hormones in ERα-positive breast cancer cells is very important to evaluate the potential threat to breast cancer patients. We observed that down-regulation of ERα could substantially block BPAF-induced endogenous transcription in ERα-positive breast cancer cells. Moreover, agent able to block ER activation such as ICI 182780 prevented *TFF1*, *GREB1* and *CTSD* induction by BPAF treatment. Overexpression of ERα in ERα-negative MDA-MB-231 human breast caner cells can significantly stimulate *TFF1*, *GREB1* and *CTSD* mRNA expression in response to BPAF. These results indicated that ERα plays a central role in regulating BPAF-induced endogenous transcription. Both E2 and BPAF could strongly stimulate 3×ERE and *TFF1* ERE luciferase reporters in HepG2 cells transiently transfected with ERβ, indicating E2 or BPAF could promote ERβ binding to ERE element to stimulate transcriptional activity [7]. In our study, BPAF-induced transcription of *TFF1*, *GREB1* and *CTSD* did not change in ERβ-silencing cells compared to the cells transfected with control siRNA, suggesting ERβ did not mediate endogenous transcription. ERα and ERβ, which act as ligand-activated transcription factors, response differently to ligands in regulating gene expression [24]. Our findings provided the evidence that endogenous transcription of estrogen responsive genes was dependent on ERα but not ERβ in human breast cancer cells.

In addition, BPAF-induced gene transcription was also partially blocked by GPER siRNA, suggesting there are other factors required to induce endogenous transcription. It is well known that E2 regulates human physiology *via* both genomic and nongenomic pathways [25], [26]. The genomic and nongenomic actions are connected to each other and regulate estrogen signaling in a coordinated fashion [27]. In the nongenomic pathway, E2 could activate GPER and trigger rapid intracellular signalings, including epidermal growth factor receptor-dependent ERK1/2 activation [28]. Therefore, we are wondering whether BPAF-induced endogenous transcription is mediated *via* a nongenomic and ERK1/2-dependent pathway. Pupo *et al* reported that BPA induces gene expression through GPER/EGFR/ERK1/2 signaling in SK-BR-3 breast cancer cells [29]. In consistence with the above study, we found that BPAF-induced mRNA expression was partially decreased by silencing GPER with siRNA, which suggested that GPER-mediated nongenomic action could take part in the endogenous transcription stimulated by BPAF. Many studies have demonstrated that E2 stimulates nongenomic action including rapid and transient activation of Src/MEK/ERK1/2 signaling [28]. Activation of this signaling triggers vital cellular functions including cell cycle control, cell proliferation and differentiation in a variety of cells and tissues [30], [31]. Similarly, we found inhibition of MEK activity by PD98059 and U0126 significantly reduced the ability of BPAF to induce the mRNA expression of *TFF1*, *GREB1* and *CTSD*, indicating ERK1/2 activation is required for BPAF-stimulated gene transcription. Moreover, inhibition of Src, which could active MEK, also blocked BPAF-induced endogenous transcription. Our results suggested that BPAF-induced gene expression is dependent on the nongenomic pathway involving the activation of GPER/Src/ERK1/2 signaling. Activation of ERK1/2 which translocates to the nucleus possibly facilities the ERα binding to the ERE located in or near promoter regions of target genes.

Thus, the precise molecular mechanism by which BPAF stimulates ERK1/2 signaling in human breast cancer cells has not been fully elucidated. It has been reported that GPER mediated the nongenomic pathway through epidermal growth factor receptor (EGFR)-dependent ERK1/2 activation in a variety of estrogen responsive cells [32]–[34]. However, EDCs appeared to activate the ERK1/2 and Src tyrosine kinase in an ERα-dependent manner [35], [36]. ERα could regulate both E2 and BPA-induced pancreatic insulin content through Src/ERK1/2 pathway [36]. Li *et al* reported that EDCs activate ERE-mediated transcriptional activation *via* ERK1/2 and Src in Ishikawa/ERα-stable cells [6]. Nevertheless, further studies are needed to better understand the role of GPER and ERα in BPAF-induced activation of ERK1/2 signaling.

In summary, we demonstrated that BPAF stimulated endogenous transcription of estrogen responsive genes through dose-dependent and time-dependent manners. Down-regulation of ERα by siRNA or inhibition of ER activity by ICI 182780 significantly inhibited BPAF-induced endogenous transcription in T47D and MCF7 cells. In contrast, overexpression of ERα in MDA-MB-231 cells resulted in enhanced gene transcription, suggesting ERα regulated BPAF-induced genes transcription in human breast cancer cells. Moreover, we found that GPER and ERK1/2 were also required to stimulate endogenous transcription. Taken together, the present study explained that both ERα-mediated genomic pathway and ERK1/2-dependent nongenomic pathway played an important role in BPAF-induced endogenous transcription of estrogen responsive genes in human breast cancer cells.

## Supporting Information

File S1 **Figures S1–S3.** Figure S1. Western blot and real-time PCR analysis of ERα, ERβ and GPER in T47D and MCF7 cells. Figure S2. Western blot and real-time PCR analysis of ERα in MDA-MB-231 cells infected with adenovirus expressing ERα (Ad-ERα) or GFP (Ad-GFP). Figure S3. BPAF-induced ERK1/2 phosphorylation in T47D and MCF7 cells.
(DOC)
