# Supplementary material for: Bisphenol AF-Induced Endogenous Transcription Is Mediated by ERα and ERK1/2 Activation in Human Breast Cancer Cells
Source: PLoS One. 2014 Apr 11;9(4):e94725. doi: 10.1371/journal.pone.0094725 (PMC3984236; doi:10.1371/journal.pone.0094725)
Supplement: File S1 — Figures S1–S3. Figure S1. Western blot and real-time PCR analysis of ERα, ERβ and GPER in T47D and MCF7 cells. Figure S2. Western blot and real-time PCR analysis of ERα in MDA-MB-231 cells infected with adenovirus expressing ERα (Ad-ERα) or GFP (Ad-GFP). Figure S3. BPAF-induced ERK1/2 phosphorylation in T47D and MCF7 cells. (DOC) [file pone.0094725.s001.doc]

**Supporting Informaiton**

**Figure S1**

**
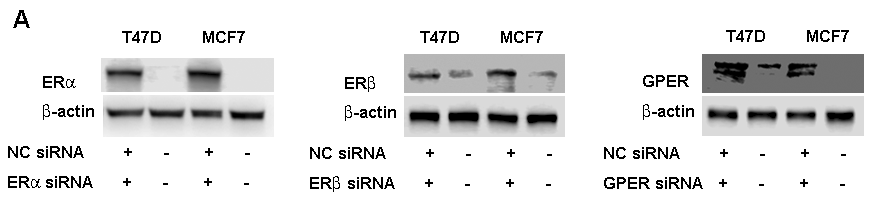
**

**
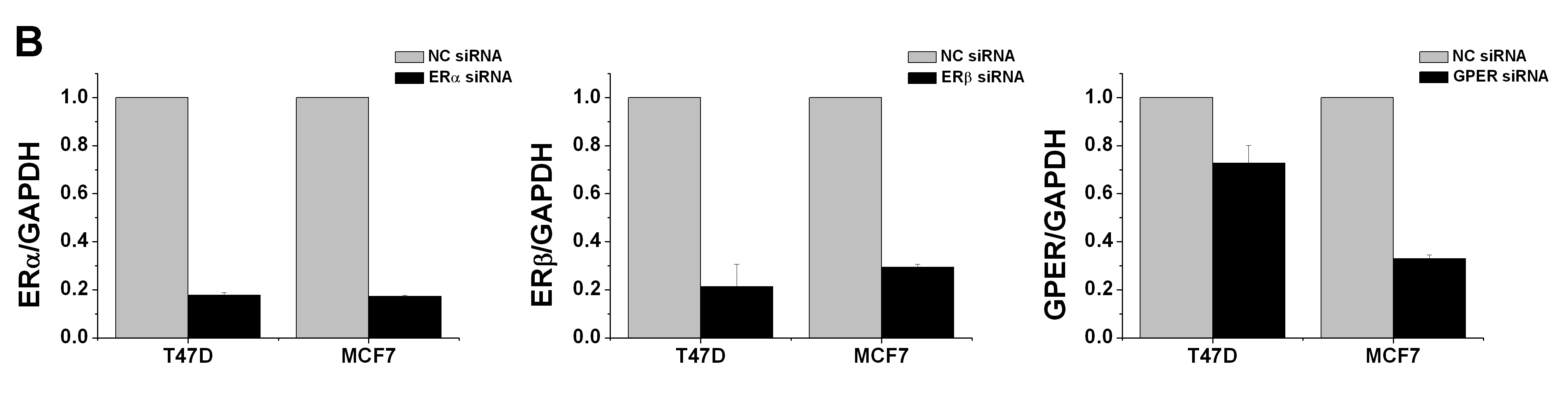
**

**Figure S1 Western blot and real-time PCR analysis of ERα, ERβ and GPER in T47D and MCF7 cells.** (A) Western blot analysis of ERα, ERβ and GPER in T47D and MCF7 cells **transfected with siRNA**. Cells were transfected with NC, ERα, ERβ and GPER siRNA for 48 h, respectively. Then, cells were harvested and cell lysate was applied to western blot. β-actin was used as a loading control. (B) Relative levels of ERα, ERβ and GPER mRNA in T47D and MCF7 cells. mRNA was detected using real-time PCR with GAPDH as an internal control.

**Figure S2**


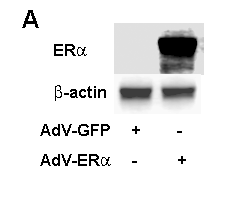

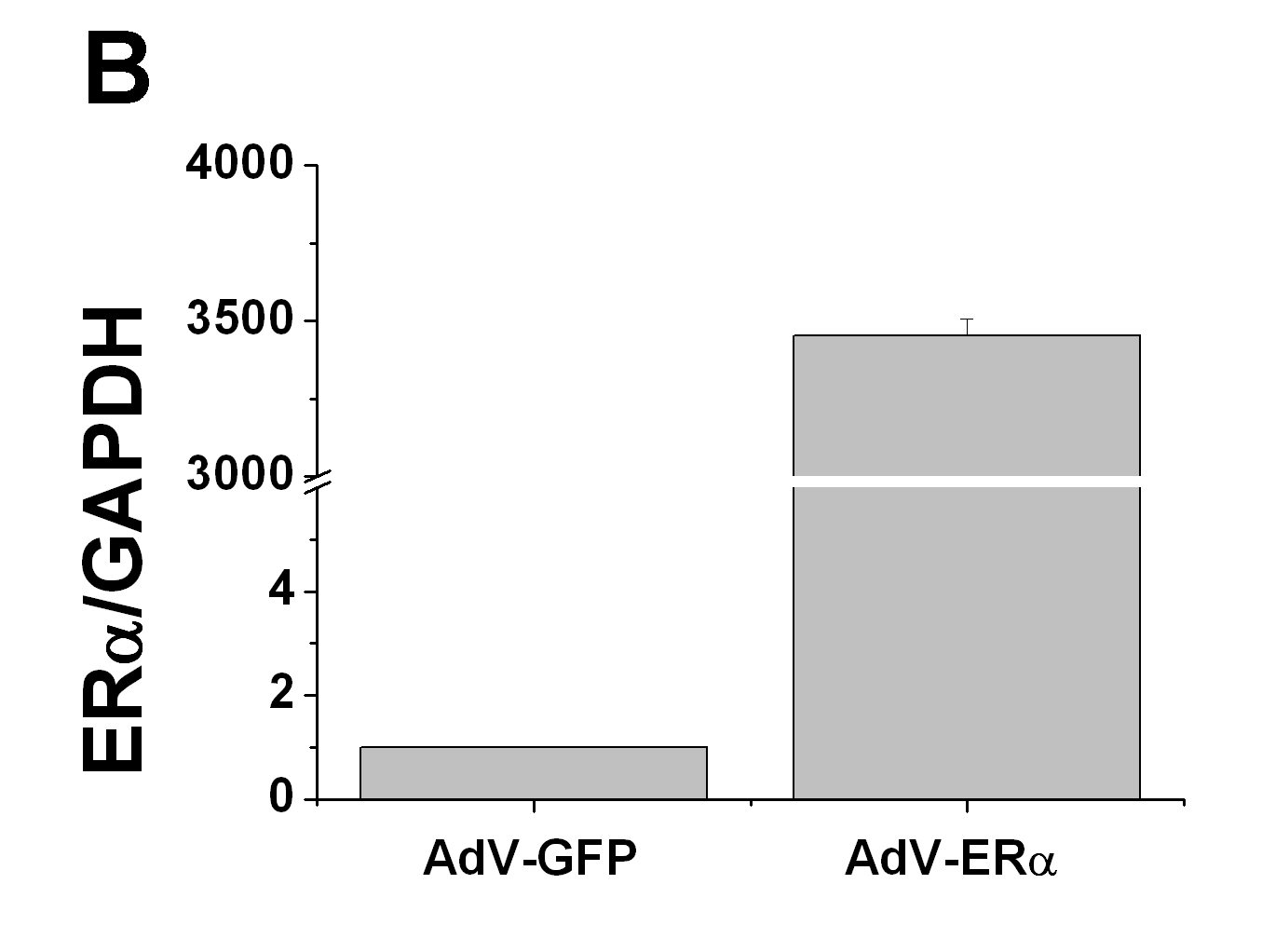


**Figure S2 Western blot and real-time PCR analysis of ERα in MDA-MB-231 cells infected with adenovirus expressing ERα (Ad-ERα) or GFP (Ad-GFP).** (A) Western blot analysis of ERα in MDA-MB-231 cells. MDA-MB-231 cells were infected with Ad-GFP and Ad-ERα, respectively. Cells were harvested at 24 h after infection. Cell lysate was applied to western blot. β-actin was used as a loading control. (B) Relative level of ERα in MDA-MB-231 cells. mRNA was detected using real-time PCR with GAPDH as an internal control.

**Figure S3**

**
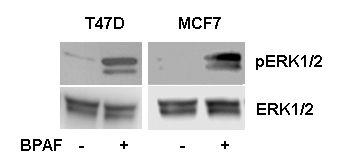
**

**Figure S3 BPAF-induced ERK1/2 phosphorylation in T47D and MCF7 cells.** T47D and MCF7 cells were stimulated by treatment with 1 µM BPAF for 15 min. The phosphorylation and total of ERK1/2 was detected by western blot.
